# Mild Intrauterine Hypoperfusion Leads to Lumbar and Cortical Hyperexcitability, Spasticity, and Muscle Dysfunctions in Rats: Implications for Prematurity

**DOI:** 10.3389/fneur.2018.00423

**Published:** 2018-06-15

**Authors:** Jacques-Olivier Coq, Maxime Delcour, Yuko Ogawa, Julie Peyronnet, Francis Castets, Nathalie Turle-Lorenzo, Valérie Montel, Laurence Bodineau, Phillipe Cardot, Cécile Brocard, Sylvie Liabeuf, Bruno Bastide, Marie-Hélène Canu, Masahiro Tsuji, Florence Cayetanot

**Affiliations:** ^1^Centre National de la Recherche Scientifique, Institut de Neurosciences de la Timone, UMR 7289, Aix Marseille Université, Marseille, France; ^2^Centre National de la Recherche Scientifique, Neurosciences Intégratives et Adaptatives, UMR 7260, Aix Marseille Université, Marseille, France; ^3^Department of Regenerative Medicine and Tissue Engineering, National Cerebral and Cardiovascular Center, Suita, Japan; ^4^Centre National de la Recherche Scientifique, Institut de Biologie du Développement de Marseille, UMR 7288, Aix-Marseille Université, Marseille, France; ^5^FR 3512 Fédération 3C, Aix Marseille Université – Centre National de la Recherche Scientifique, Marseille, France; ^6^EA 7369 ≪Activité Physique, Muscle et Santé≫ - URePSSS - Unité de Recherche Pluridisciplinaire Sport Santé Société, Université de Lille, Lille, France; ^7^Institut National de la Santé et de la Recherche Médicale, UMR_S1158 Neurophysiologie Respiratoire Expérimentale et Clinique, Sorbonne Université, Paris, France

**Keywords:** neonatal hypoxia-ischemia, cerebral palsy, intrauterine growth retardation, white matter injury, KCC2

## Abstract

Intrauterine ischemia-hypoxia is detrimental to the developing brain and leads to white matter injury (WMI), encephalopathy of prematurity (EP), and often to cerebral palsy (CP), but the related pathophysiological mechanisms remain unclear. In prior studies, we used mild intrauterine hypoperfusion (MIUH) in rats to successfully reproduce the diversity of clinical signs of EP, and some CP symptoms. Briefly, MIUH led to inflammatory processes, diffuse gray and WMI, minor locomotor deficits, musculoskeletal pathologies, neuroanatomical and functional disorganization of the primary somatosensory and motor cortices, delayed sensorimotor reflexes, spontaneous hyperactivity, deficits in sensory information processing, memory and learning impairments. In the present study, we investigated the early and long-lasting mechanisms of pathophysiology that may be responsible for the various symptoms induced by MIUH. We found early hyperreflexia, spasticity and reduced expression of KCC2 (a chloride cotransporter that regulates chloride homeostasis and cell excitability). Adult MIUH rats exhibited changes in muscle contractile properties and phenotype, enduring hyperreflexia and spasticity, as well as hyperexcitability in the sensorimotor cortex. Taken together, these results show that reduced expression of KCC2, lumbar hyperreflexia, spasticity, altered properties of the soleus muscle, as well as cortical hyperexcitability may likely interplay into a self-perpetuating cycle, leading to the emergence, and persistence of neurodevelopmental disorders (NDD) in EP and CP, such as sensorimotor impairments, and probably hyperactivity, attention, and learning disorders.

## Introduction

It is now well admitted that perinatal brain damage and neurodevelopmental disorders (NDD) are usually related to several conditions, such as neonatal encephalopathy, perinatal arterial ischemic stroke, systemic infections, and premature birth (1). In the worst cases, perinatal brain damage often leads to cerebral palsy (CP), which is a complex syndrome of various sensory, motor (including spasticity, contractures, and spams), and cognitive deficits and is considered the main cause of physical disability in children (2, 3). About 40% of extremely born preterm children (24–32 weeks of gestation) develop moderate to severe sensorimotor and/or cognitive impairments, while the rest of these very preterm children often exhibit minor motor, behavioral, and cognitive disorders. Premature birth occurs in 1/8 of deliveries, but the proportion of prematurity is steadily increasing since the early 1990s in developed countries (1, 4). With increasing prevalence, encephalopathy of prematurity (EP) is mainly characterized by gray matter dysmaturation and reduction, diffuse white matter injury (WMI) related to abnormal oligodendroglial precursor maturation leading to hypomyelination, minor to mild sensorimotor, behavioral and cognitive disorders, and often results in CP (1). Although the relations between the NDDs, brain damage, perinatal hypoxia-ischemia, and neuroinflammation are not completely clear and understood (3, 5), we recently developed a rodent model of EP, based on prenatal ischemia (6) which in fact better corresponds to mild intrauterine hypoperfusion (MIUH) (7).

In a prior series of studies (8–10), we used prenatal ischemia or MIUH at embryonic day 17 (E17), considered to be equivalent to embryonic weeks 20–25 in humans (11, 12). MIUH at E17 led to myelination deficits in the corpus callosum and cingulum of rat neonates when assessed between birth and postnatal day 21 (P21) (13, 14). In rats examined at adulthood after MIUH at E17, hypomyelination and axonal degeneration persisted in the internal and external capsules, corpus callosum, fornix, pontocerebellar tract, and in white matter zones below the cingular and primary somatosensory cortices. No hypomyelination nor axonal degeneration were detected in white matter zones below the primary motor cortex or in the corticospinal tract. Massive astrogliosis was also observed in white matter associated with the somatosensory cortex, as well as enlargement of the lateral ventricules (7–10). Interestingly, the severity of hypomyelination in these adult rats correlated with the gradient of growth restriction at birth (9, 13, 14). The cerebral area in MIUH rats was reduced at striatal and hippocampal levels at P15 (7). We also found decreased neuronal densities in the somatosensory cortex, particularly inhibitory neurons, and decreased height of the cortical gray matter in adult MIUH rats. In contrast, there was no structural changes in the motor cortex (9). In addition, abnormal lamination of the parietal cortex, presumably due to premature disruption of the cortical subplate, was associated with gait disturbances in adult rats exposed to MIUH at E17-18 (12, 15). There was no obvious tissue damage, such as cystic and necrotic lesions or inflammatory cell infiltration in adult MIUH rats (7). As a neonatal index of sensorimotor reflexes, negative geotaxis was delayed in MIUH pups. P15 and adult MIUH rats exhibited spontaneous exploratory and motor hyperactivity. Adult MIUH rats displayed deficits in information encoding, and deficits in short and long term object memory tasks, but no impairments in spatial learning or working memory in watermaze tasks (7, 9, 10). These behavioral and cognitive deficits in our rodent model appear to recapitulate some symptoms commonly found in children with EP, such as attention-deficit with hyperactivity disorder (ADHD), and learning and memory deficits (4, 16, 17). In addition to neuroanatomical disturbances, the primary somatosensory maps representing the hind paw skin surfaces were topographically disrupted and disorganized in MIUH rats, compared to controls (10), likely indicative of reduced tactile abilities (18). In contrast, we found no changes in the neuroanatomical and functional organization of the primary motor cortex (10). Adult MIUH rats exhibited minor locomotor deficits on treadmill with mainly knee-ankle hyperextension compensated by hip hyperflexion, as well as increased variations in locomotor kinetics that appeared mainly related to a disorganization in the somatosensory cortex but not in the motor cortex (8, 10), as observed in children with CP (19). MIUH rats also displayed muscle weakness in their hind limbs but not in their forelimbs, mild myopathic and secondary joint changes in their hind limbs, indicative of mild signs of spasticity (7, 8) that still remain to confirm.

From the previous studies related to MIUH cited above, we wondered what may relate the various MIUH-induced events such as inflammation, WMI, sensorimotor network disorganization, minor locomotor impairments and the emergence of NDDs. We suppose that MIUH-induced intrauterine inflammation induces early and postnatal pathophysiological cascades that may involve KCC2. KCC2, a K-Cl cotransporter is the main chloride extrusion system in the central nervous system (CNS) and regulates chloride homeostasis and neuronal excitability (20–23). Another candidate in this pathophysiological cascade is the disruption of the neuromuscular interactions, especially early, and long-lasting changes in stretch reflex and muscle properties/phenotype. We hypothesized that early inflammation leads to decreased expression of KCC2, which in turn may drive spinal and cortical hyperexcitability, altered neuromuscular interactions and muscle properties, thus inducing a disorganization of the sensorimotor circuitry and locomotor impairments, and subsequent NDDs. The present study was aimed at investigating the early and long-lasting mechanisms that may contribute to the disorganization of the CNS and to the subsequent emergence of locomotor impairments and NDDs in rats after MIUH. Such a better understanding of the early pathophysiological cascades that lead to EP and CP may allow us to develop new strategies of remediation and prevention.

## Materials and methods

All experiments and animal use have been carried out in accordance with the guidelines laid down by NIH (NIH Publication #80-23) and EC Council Directive (2010/63/EEC). The research involving animals has been approved by the local ethics committees in Marseille (Comité d'éthique en Neurosciences INT-Marseille—CEEA #71, authorization #00265.02), Lille (Comité d'éthique Région Nord Pas-de-Calais—CEEA #75, authorization APAFIS#4732-2016031112395755), and Japan (Committee of the National Cerebral and Cardiovascular Center, Suita, Japan).

### Intrauterine arterial strenosis using microcoils

**Figure 1:**
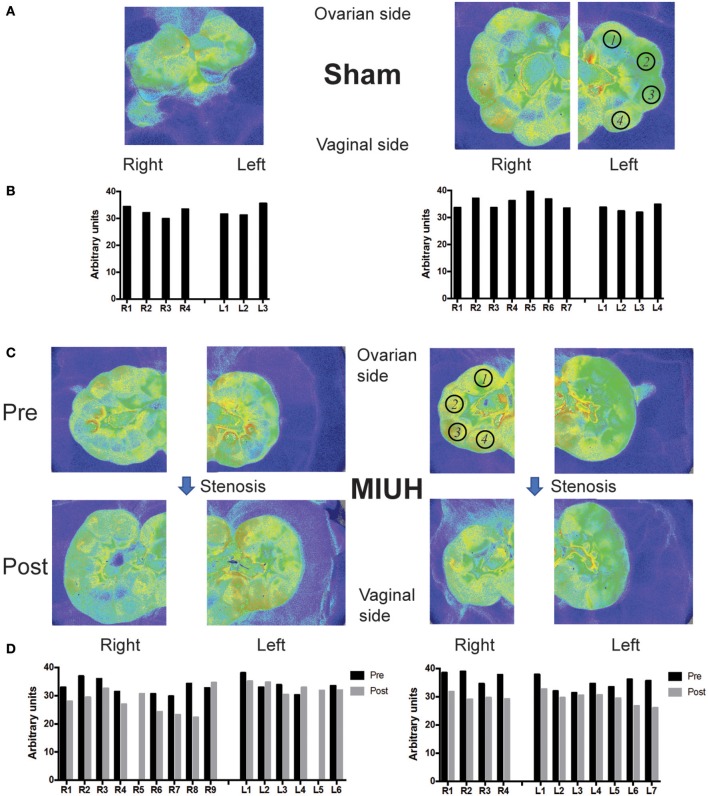
Laser speckle flowmetry and blood flow measures in the fetuses and placentas of pregnant rats that experienced either sham operation or microcoil stenosis at embryonic day 17 (E17) leading to mild intrauterine hypoperfusion (MIUH). **(A)** Images of laser speckle of the uterine horns in two representative sham rats at E17. **(B)** Quantification of blood flow in fetuses that correspond to the laser speckle images in **(A)**. **(C)** Images of laser speckle showing changes in uterine blood flow before (Pre) and 1 h after microcoil stenosis (Post) at both sides of the ovarian artery. **(D)** Blood flow changes during coils stenosis in fetuses in both dams shown in **(C)**. The blood flow decreased 1 h after stenosis (Post) compared to that before stenosis (Pre) in most of the fetuses and placentas (not shown). The location of pups in the right (R) and left (L) horns of the uterus is denoted from 1 to the maximal number of pups in each side, with 1 at the closest location to the ovary and the last one at the closest location to the vagina. The region of interest (ROI) for blood flow measures of the fetuses is depicted in **(A,C)** by black open circles, with numbers that correspond to the locations of the fetus on each uterus horn.

In prior studies, the intrauterine artery stenosis was performed by using unilateral ligation at embryonic day 17 (E17) to produce prenatal ischemia and intrauterine growth retardation in rats (8–10, 13). We developed a new model of intrauterine ischemia/growth retardation based on the application of metal-coated coils (Samini Co. Ltd., Shizuoka, Japan) wrapped around the intrauterine arteries at E17 (7, 24). Briefly, under deep anesthesia with isoflurane, microcoils (inner diameter: 0.16 mm) were wrapped around each proximal artery of both ovarian sides by using the same procedure as described previously (5, 6), to produce optimized blood flow reduction or MIUH (Figure 1). This technique has the advantage to increase the number of MIUH pups and thus to reduce the number of used dams, compared to stenosis by ligation. The sham group was subjected to the same surgery as the MIUH group but without coil insertion. Pups were delivered by spontaneous labor and attributed to each group depending on the weight at birth. Like in previous studies, pups whose weight was below 5.5 g were considered growth retarded and part of the MIUH group.

### Laser speckle blood flowmetry

Temporal changes of intrauterine blood flow during the surgery were monitored by using a laser speckle flowmetry (Omegazone, Omegawave Inc., Tokyo, Japan) at two time points: before stenosis and 1 h after stenosis on both ovarian and vaginal sides (Figure 1) under isoflurane deep anesthesia. To quantify blood flow, regions of interest (ROI) on all fetuses or placentas were analyzed in 2 sham and 2 MIUH rats (Figures 1A,C).

### Early *in vitro* post-activation depression

To determine the early impact of MIUH on the functional reorganization and excitation/inhibition balance in rat pups, we assessed the alterations of the monosynaptic reflex loop using an *in vitro* whole spinal cord preparation at postnatal days P4–P6 (Sham, *n* = 11; MIUH, *n* = 6). The spinal cord below T8 was isolated from neonatal rats from P4 to P6, as previously described (21, 22) and transferred to the recording chamber perfused with an oxygenated (95% O_2_/5% CO_2_) aCSF composed of the following (in mM): 130 NaCl, 4 KCl, 3.75 CaCl2, 1.3 MgSO_4_, 0.58 NaH_2_PO_4_, 25 NaHCO_3_, and 10 glucose (pH 7.4; 32°C). Extracellular recordings/stimulation were made at lumbar L5 ventral (VR5) and dorsal (DR5) roots by contact stainless steel electrodes insulated with vaseline. AC recordings from VR were amplified (×2,000) and bandpass filtered from 70 Hz to 3 kHz. Supramaximal stimulation of a lumbar dorsal root (DR5) elicited a monosynaptic response in the ipsilateral homonymous ventral root (VR5) *in vitro*, corresponding to the earliest component of motoneurons excitation. To determine the level of post-activation depression (PAD) at the different frequencies, we discarded responses to the first three stimulations required for the depression to occur. The responses were rectified and the areas under the curves were measured. The monosynaptic response was expressed as percentages relative to the mean response at 0.1 Hz in the same series of measurements (21).

### Postnatal KCC2 western blots

To detect the expression of KCC2 in the spinal cord, tissue was collected at P8 and frozen after removing the dorsal and ventral roots (Sham, *n* = 8; MIUH, *n* = 7). Samples were prepared in ice–cold lysis buffer containing 1% Igepal CA-630, 0.1% SDS, 10 mM sodium vanadate, 10 mM sodium fluoride, 10 mM sodium pyrophosphate, 1.8 mg.mL^−1^ iodoacetamide supplemented with protease inhibitors cocktail (Complete-mini, Roche Life Science). After centrifugation step at 18,000 g for 30 min at 4°C, the supernatant was collected, and protein concentrations were determined using DC protein assay (Biorad). Equal protein amounts (30 μg) from samples were separated by electrophoresis SDS-PAGE (4-15% Criterion™ TGX Stain-Free™ Precast Gels, Biorad), transferred to a nitrocellulose membrane and incubated overnight at 4°C with the affinity-purified rabbit anti-KCC2 polyclonal antibody (diluted 1:1,000; Merck-Millipore). The blot was then incubated 1 h at 24°C with an immunoPure goat HRP-conjugated rabbit specific antibody (1:80,000; Thermo Scientific, in blocking solution of Tris buffered saline containing 5% fat-free milk powder). Bands were visualized by chemiluminescence (Merck-Millipore). Signal intensities were measured with the image analysis software Labview (BioRad). Equal amounts of protein samples were loaded, and we performed total protein normalization using stain-free imaging (Biorad), which makes proteins fluorescent directly in the gel and following transfer (see Supplemental Figure 1). The total density for each lane was measured from the blot and was used to calculate the normalizing factors. After normalization to total proteins of signal intensity for KCC2, we normalized by dividing each sample by the mean value of control samples.

### Adult *in vivo* post-activation depression

The Hoffmann reflex is commonly used to assess primary (type Ia) afferents–mediated motor neuronal excitability (monosynaptic reflex loop) in individuals with spasticity 21, 25. The H-reflex was measured in P60 rats from both groups (Sham, *n* = 9; MIUH, *n* = 8) under deep and constant anesthesia, induced first with isoflurane and then with ketamine (100 mg.Kg^−1^ i.p. induction) and maintained with Supplemental doses of ketamine (20 mg.Kg^−1^ i.p.), as widely used (21, 23, 26). Rat temperature was maintained around 38°C with a thermal pad controlled by rectal temperature probe. A transcutaneous pair of stainless stimulating needle electrodes was inserted adjacent to the tibial nerve about 1 cm above the ankle. For EMG, a pair of stainless recording electrodes was inserted into the flexor digitorum beneath the ankle and the reference electrode into the tail's skin. First, we stimulated the tibial nerve for 0.2 ms at 0.2 Hz with increasing current intensities until the M_max_ stabilized, and determined the intensity required for a maximal H response. Tibial nerve was stimulated with trains of 20 stimulations at 0.2, 0.5, 1, 2, and 5 Hz with 2 min intervals between each train to elicit PAD. To determine the level of PAD at the different frequencies, we discarded responses to the first three stimulations required for the depression to occur. The M and H waves were rectified and the areas under the curves were measured. The H responses were expressed as percentages relative to the mean response at 0.2 Hz in the same series of measurements (21, 23).

### *In situ* contractile properties of the soleus muscle and muscle removal

At P28, the rats were deeply anesthetized with intraperitoneal injections of ketamine (50 mg.Kg^−1^) and dexmedetomidine (Domitor, 0.25 mg.Kg^−1^), prolonged if necessary by Supplemental doses. The dissection protocol was previously described (27). Briefly, all the muscles of the right hindlimb were denervated, except the soleus muscle, which was isolated from surrounding tissues. Then, the limb was immersed in a bath of paraffin oil thermostatically controlled (37°C), and fixed with bars and pins. The soleus muscle was maintained in a horizontal position and its distal tendon was connected to a force transducer (Grass FT 10; Grass Instruments, West Warwick, RI, USA). The muscle length was adjusted to produce a maximal twitch peak tension (P_t_). Contractions were induced by stimulation of the sciatic nerve (0.2-ms pulses) through bipolar platinum electrodes at twice the minimum voltage required to obtain the maximal twitch response. The following parameters were recorded: P_t_, time-to-peak (TTP), half-relaxation time (HRT); peak tetanic tension obtained at 100 Hz (P_0_). The fatigue index (FI) was calculated as the percent of the initial tension divided by the force at the end of the fatigue protocol in a series of 120 consecutive contractions (330 ms duration, 40 Hz, one train per second). At the end of the recording session, the muscle was removed for determination of the soleus muscle wet weight (MWW), frozen in liquid nitrogen and stored at −80°C until electrophoretic analysis (see Supplemental Methods). This study was performed on 17 pups (Sham, *n* = 8; MIUH, *n* = 9).

### Excitatory and inhibitory neurotransmission

To gain insights into the long-term impact of MIUH on the extra- and intracellular levels of glutamate and GABA, we performed *in vivo* microdialysis within the sensorimotor cortex contralateral to the mapped side from P90 to 120. In other rats, Western blot analysis was used to quantify the intracellular amounts of transporters of both glutamate (vGLUT1) and GABA (vGAT) in sensorimotor cortex tissues collected contralateral to the mapped side (Sham, *n* = 13; MIUH, *n* = 16).

#### *In vivo* microdialysis

Carnegie Medecin microdialysis probes (CMA/11, Phymep, France) were implanted within the hindpaw representation of the right S1-M1 area using stereotaxic coordinates (A: −1/0, L: +2; H: 2); the left cortex was mapped. The chosen implantation site of the probe avoided large blood vessels; traces of blood were not found after probe withdrawal. The terminal ends of the probes were covered with a polycarbonate membrane. The membrane had a diameter of 0.24 mm, a length of 2 mm to allow to sample all cortical layers and a 6 KDa molecular mass cut-off. The membrane acted like a blood vessel, with extracellular molecules passing through it by diffusion gradient. A new probe was used for each rat. This procedure is detailed in Supplemental Methods.

#### Western blotting and quantification

The leg region from the right side of the sensorimotor cortex was collected and homogenized in TBS (Tris-HCl 50 mmol.L^−1^ pH 7.4, NaCl 150 mmol.L^−1^) containing protease inhibitors (complete EDTA free, Roche, Basel, Switzerland) using a Potter homogenizer and the ratio of 100 μg of brain pieces per mL of buffer. Homogenates were centrifuged at 500 g for 3 min at 4°C. Supernatants were aliquoted and stored at −80°C. Proteins present in each supernatant were resolved by SDS-PAGE and transferred onto nitrocellulose membranes (see Supplemental Methods and Supplemental Figure 2). Proteins of interest were then detected with specific antibodies using chemiluminescence (ECL; Pierce Biotechnology, Rockford, IL, USA). Western blot quantification was performed on scanned autoradiographies with Image J software (28). Integrative intensities minus background were plotted for each sample. Values were normalized to 1 for the highest value for one western blot.

## Data analysis

Data normality and homogeneity of variance were determined with the Shapiro test, Bartlett test and var.test by using R (The R Foundation for Statistical Computing, Wien, Austria). We then applied either parametric (two-tailed and paired *t*-tests, and one-way ANOVAs with Tukey's *post-hoc* comparisons and two-way ANOVAs) or non-parametric (Mann-Whitney, Wilcoxon and ANOVA of Friedman) tests using either R or Prism (GraphPad Software, CA). Significance was set at *p* < 0.05. The investigators were blind to rearing conditions throughout the different experimental sessions until statistical comparisons were performed.

## Results

### Coil stenosis induces a mild intrauterine hypoperfusion

Our preliminary results showed that microcoil stenosis of the ovarian artery at both ovarian sides decreased the blood flow in fetuses to 17.4% and placentas to 15.3% of pre-stenosis level in average (Figures 1C,D). The level of blood supply was mostly the same across each fetus (arbitrary unit of blood flow measurement: 33.7 ± 0.8 – mean ± SEM) and each placenta (33.1 ± 1.4) before stenosis (Figures 1C,D), and compared to sham conditions (fetus: 34.9 ± 0.7; placenta: 30.9 ± 1.2; Figures 1A,B). Applied to the bilateral ovarian branches of the uterine artery at E17, our new technique of microcoil stenosis produced a consistent and reproducible MIUH across all the placentas and fetuses. The hypoperfusion apparently resolved within 3 days at E20 [data not shown, see (7)].

### MIUH leads to early hyperreflexia

**Figure 2:**
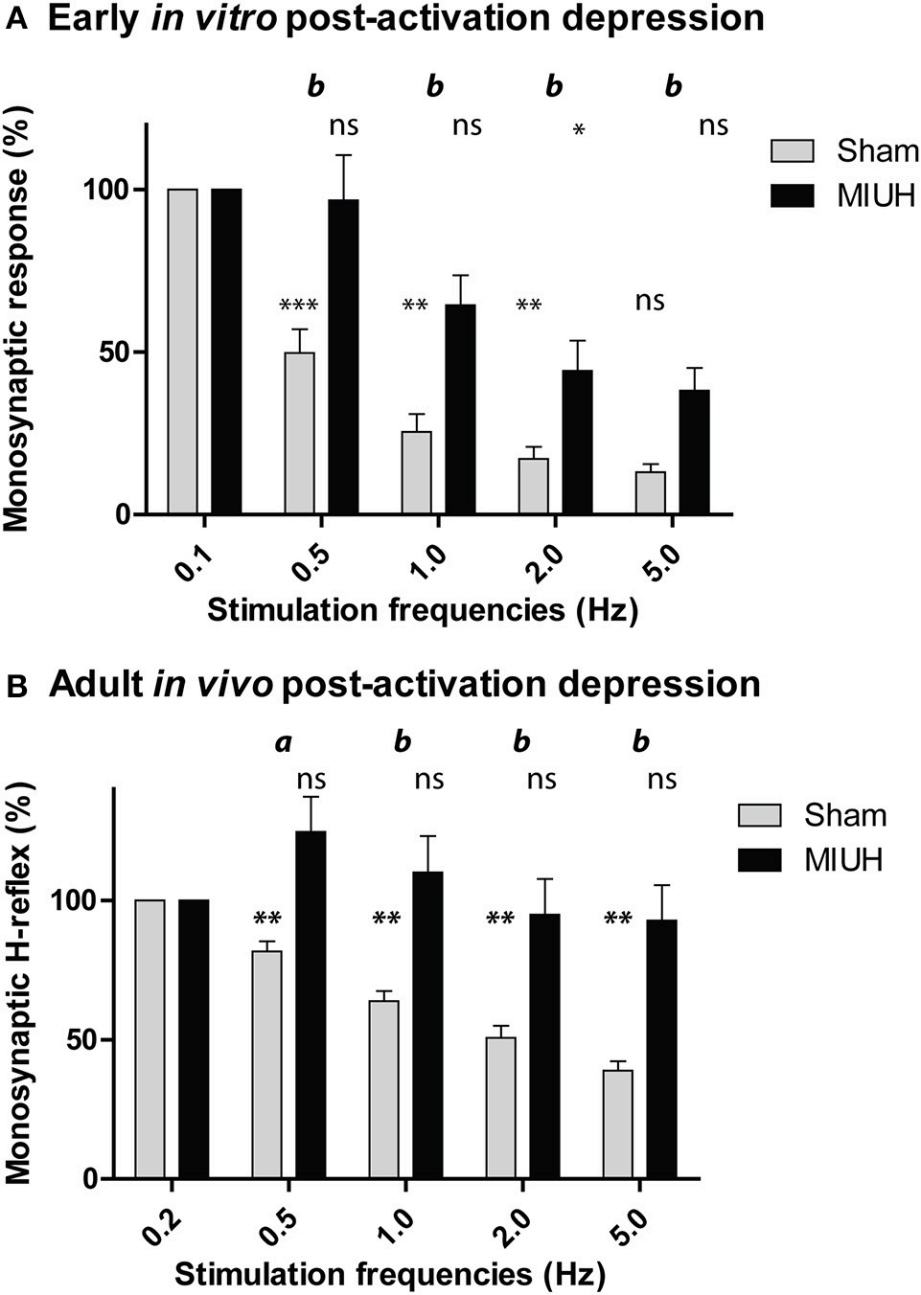
Decreased post-activation depression (PAD) performed *in vitro* in pups from P4 to P6 and *in vivo* in adult rats that both were submitted to mild intrauterine hypoperfusion (MIUH) at E17. **(A)** Mean (± SEM) relative amplitudes of the monosynaptic reflex at different stimulation frequencies in MIUH pups, compared to sham rats. In sham rats, the monosynaptic amplitude decreased with increasing frequencies (i.e., PAD) from 0.1 Hz (set as reference = 100%) to 5 Hz [(ANOVA of Friedman, x^2^ = 40.1, df = 4; *p* < 0.0001) and Wilcoxon's *post-hoc* comparisons:^**^p < 0.01; ^***^p < 0.0001; ns, non-significant] while this amplitude decreased lesser in MIUH pups [(ANOVA of Friedman, x^2^ = 21.1, df = 4; *p* < 0.003) and Wilcoxon's *post-hoc* comparisons:^*^p < 0.05; ns, non-significant]. The comparison of the monosynaptic reflex amplitude between sham and MIUH rats showed significant reduction of the PAD at stimulation frequency (Wilcoxon: *b, p* < 0.01) from 0.1 Hz. **(B)** Depression of the H-reflex (PAD), expressed as percentages, over consecutive stimulations from 0.2 Hz (set as reference = 100%) to 5 Hz in sham rats [(ANOVA of Friedman, x^2^ = 69.6, df = 4; *p* < 0.0001) and Wilcoxon's *post-hoc* comparisons:^**^p < 0.01] while the H-reflex was increased at 0.5 and 1 Hz and did not reduce at higher frequencies of stimulation in MIUH rats [(ANOVA of Friedman, x^2^ = 12.5, df = 4; *p* < 0.01) and Wilcoxon's *post-hoc* comparisons: ns, non-significant]. When we compared the H-reflex PAD between sham and MIUH rats, we found significant decreases of PAD at stimulation frequencies from 0.2 to 5 Hz (Wilcoxon: *a, p* < 0.05; *b, p* < 0.01) in the latter group.

In sham pups, the amplitude of the monosynaptic response decreased when the dorsal root was stimulated repeatedly. From P4 to P6, this effect became stronger with increasing stimulation frequency from 0.1 to 5 Hz (*p* < 0.0001; Figure 2A), similar to the *in vitro* PAD observed previously (21–23). In contrast, MIUH pups at P4–P6 showed a decrease in the response amplitude with increasing stimulation frequencies, but much lesser than in sham rats (*p* < 0.003). Compared to sham rats at each frequency, MIUH pups showed a significant reduction of the PAD at P4–P6 (Figure 2A), suggestive of an early increase of stretch reflex and hyperexcitability within the lumbar spinal cord and early signs of spasticity as well, as shown in pups after complete spinal cord section (21, 23).

### MIUH decreases the early amounts of KCC2

**Figure 3:**
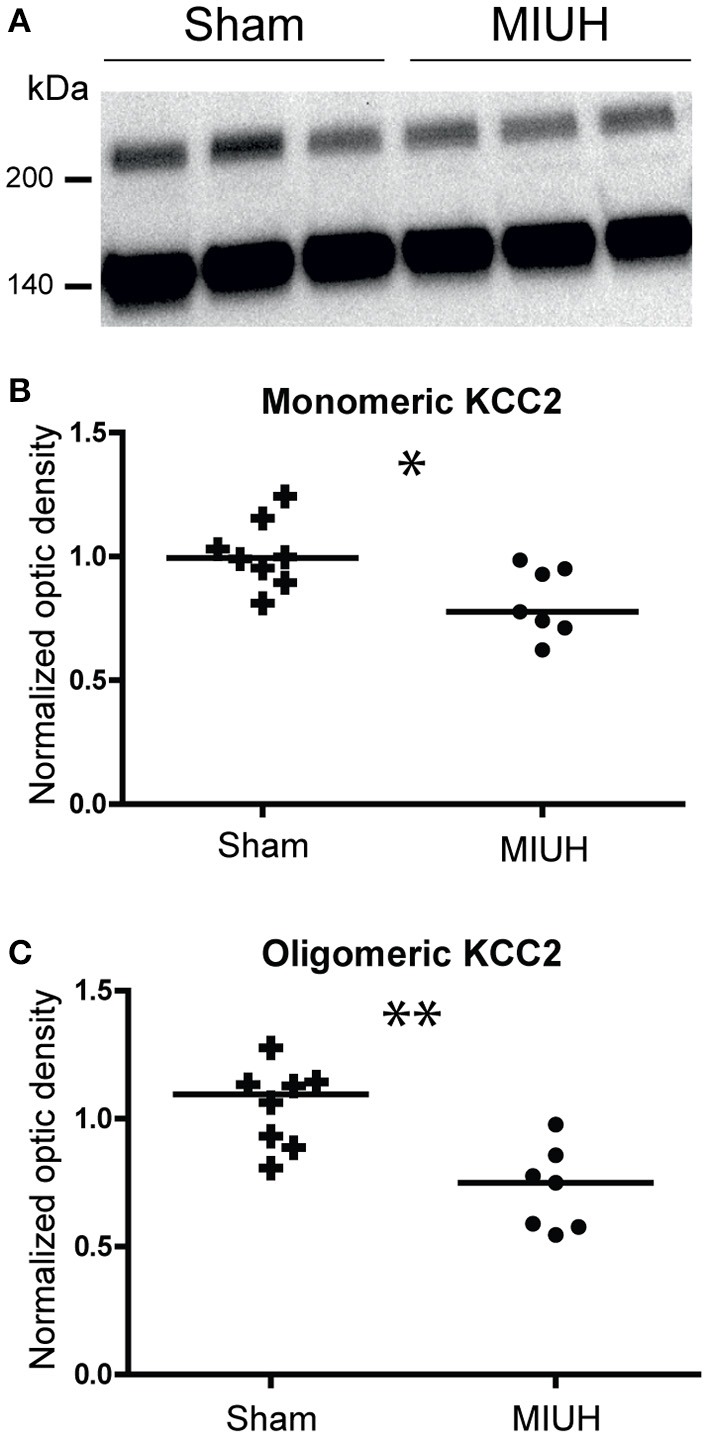
Western blotting of the monomeric and oligomeric forms from the total fraction of KCC2 in sham and MIUH rats at P8. **(A)** Typical immunoblots for the two forms of KCC2 in both groups. Left scales depict the molecular weight expressed in kDa. **(B)** Plots of the monomeric (inactive) form of KCC2 at P8. The mean quantity of monomeric KCC2 was decreased in MIUH rats compared to sham rats (*U* = 7; *p* < 0.02). **(C)** Plots of the oligomeric (active) form of KCC2 at P8. The mean quantity of oligomeric KKC2 was also decreased in MIUH rats relative to sham rats, but in a greater extent (*U* = 4; *p* < 0.04) than for the monomeric form in **(B)**. ^*^ *p* < 0.05; ^**^ *p* < 0.01.

Protein analysis was performed on the whole lysate (total fraction) from the lumbar spinal cord at P8, followed by immunoblotting with specific antibodies against KCC2 protein. The monomeric and oligomeric forms of KCC2 were detected at 140 KDa and upper than 250 KDa, respectively (See Supplemental Figure 1). Compared to sham rats, the amount of KCC2 in the total fraction decreased in MIUH rats (*p* < 0.006; data not shown). More interestingly, the monomeric and oligomeric forms were reduced after MIUH, especially the latter (*p* < 0.04; Figure 3). Monomeric and oligomeric forms correspond to the inactive and active forms of KCC2, respectively (20). This result suggests a decrease in both the active and inactive forms of KCC2 after MIUH, especially the former, responsible for chloride homeostasis and cellular excitability (20, 21).

### MIUH leads to enduring hyperreflexia in adulthood

To evaluate the persistence of spasticity in adulthood after MIUH, corresponding to hyperexcitability in the spinal circuitry, we assessed the changes in the Hoffmann reflex (H-reflex; Figure 2B) (21, 23, 29). In adult sham rats, the H-reflex was depressed by repeated nerve stimulation at frequencies increasing from 0.2 to 5 Hz (*p* < 0.0001; Figure 2B), corresponding to PAD; (23, 21), as observed above in MIUH rats at P4–P6. In contrast, the PAD was reduced in adult MIUH rats (*p* < 0.0001), as illustrated by the lack of significant differences of the monosynaptic H-reflex with increasing frequencies (Figure 2B). Surprisingly, the monosynaptic H-reflex even tended to increase at 0.5 and 1 Hz, compared to the reference at 0.1 Hz (Figure 2B). When we compared the H-reflex PAD between sham and MIUH rats, we found significant decreases with increasing frequencies from 0.2 to 5 Hz (Figure 2B), suggesting the enduring presence of hyperreflexia and spasticity in adult MIUH.

### MIUH alters adult contractile properties of the soleus muscle

At P28, no significant difference was observed in morphological parameters between sham and MIUH rats. The body weight (BW) was near 90 g in sham and MIUH groups. It should be noted that soleus muscle tended to be smaller in MIUH rats (muscle wet weight, MWW: −10%, *p* = 0.09, tendency; Table 1). However, when MWW was normalized to BW, the value was similar in both groups.

**Table 1:** Contractile and morphological properties of soleus muscles in sham and MIUH rats.

	**Sham**	**MIUH**
	**(*****n*** = **8)**	**(*****n*** = **9)**
**CONTRACTILE PROPERTIES**		
TTP (ms)	49 ± 8	52 ± 8
HRT (ms)	54 ± 4	44 ± 4^***^
P_20_/P_0_ (%)	48 ± 5	50 ± 10
P_t_ (mN)	8.03 ± 1.44	6.31 ± 1.34^*^
P_0_ (mN)	61.35 ± 5.92	55.56 ± 11.39^†^
P_t_/MWW(mN/mg)	0.22 ± 0.05	0.18 ± 0.03^†^
P_0_/MWW (mN/mg)	1.70 ± 0.36	1.49 ± 0.28
P_t_/P_0_	12.41 ± 1.22	12.10 ± 1.58
FI (%)	86 ± 9	78 ± 26
**MORPHOLOGICAL PARAMETERS**		
BW (g)	95 ± 7	89 ± 11
MWW (mg)	38 ± 4	34 ± 4
MWW/BW (mg/g)	0.40 ± 0.05	0.40 ± 0.04
**HISTOCHEMICAL PROPERTIES**		
MHC 1	67.1 ± 13.3%	55.8 ± 12.7%
MHC2A	32.2 ± 12.5%	40.3 ± 11.9%
MHC2B	0.2 ± 0.4%	0.0 ± 0.0%
MHC neonatal	0.5 ± 1.4%	3.9 ± 2.7%^**^

P_t_, single twitch tension; P_0_, tetanic tension at 100 Hz; P_20_, tetanic tension at 20 Hz; FI, fatigue index; BW, body weight; MWW, muscle wet weight.

*, **, *** A significant difference with respect to sham rats at p < 0.05, p < 0.01, p < 0.001, respectively.

† *A tendency (p < 0.1). Mean ± SD*.

The mechanical properties of the soleus muscle observed *in situ* revealed that the soleus muscle phenotype was unchanged in MIUH rats. The rate of force development was similar, as was the ratio of subtetanic tension at 20 Hz relative to P_0_ (P_20_/P_0_), which was about 50% in both groups (Table 1). This parameter is an indicator of muscle type; a low value (0.20–0.30) is characteristic of a fast muscle, whereas a high value (0.70-0.80) indicates a slow muscle. Interestingly, the interval from peak tension to half peak tension (HRT) was reduced by 18% in MIUH rats (*p* < 0.001; Table 1).

Single twitch tension (P_t_) was decreased by 21% (*p* = 0.02) in MIUH rats (Table 1). This change may due in part to the muscle atrophy, since the decrease was only −14% (*p* = 0.07, tendency) when P_t_ was normalized to MWW. In the same line, the maximal tetanic force P_0_ tended to decrease (−9%; *p* = 0.07, tendency; Table 1), but remained unchanged when normalized to MWW (−12%; *p*: n.s.).

The muscle was resistant to fatigue, whatever the group (fatigue index: 86 ± 9% in sham group and 78 ± 26% in MIUH group). This high resistance may be due to the fact that muscles were composed mainly of 1 and 2A MHC isoforms (Table 1). In contrast to sham rats where it was almost absent, a low proportion of neonatal MHC isoform was still present in the soleus muscle of MIUH rats at P28 (*p* < 0.01; Table 1). Thus, immature isoform of MHC persisted in the soleus of MIUH young-adult rats, compared to shams. It is worth noting that the proportion of MHC 2A isoform was 25% higher in MIUH group, while MHC 1 decreased by −17%. However, the overall variation was non-significant because of high inter-individual variations (Table 1). Such a transition from slow to fast isoform may explain the decrease in HRT after MIUH.

### MIUH induces hyperexcitability in the adult sensorimotor cortex

**Figure 4:**
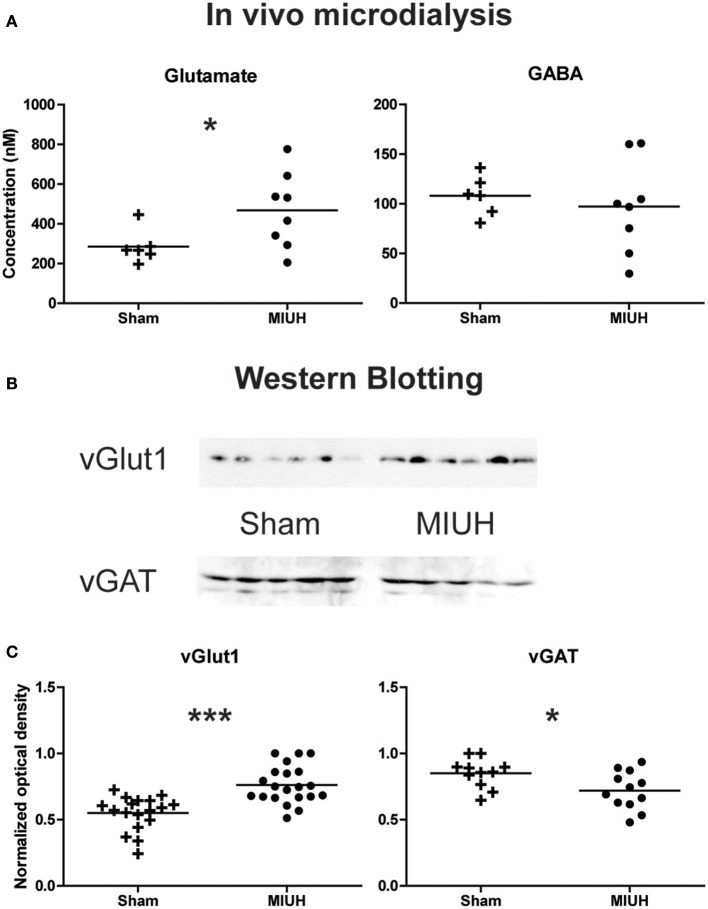
Long term effects of MIUH on the excitation/inhibition balance in the hind limb area of the sensorimotor cortex. **(A)** Plots of glutamate and GABA extracellular levels assessed by *in vivo* microdialysis in adult rats. Compared to sham rats, MIUH induced a significant increase in glutamate release (*U* = 8.0; *p* < 0.04) while the release in GABA did not differ between the two groups (*U* = 18.0; *p* = 0.4, n.s.). **(B)** Typical immunoblots for vGlut1, vesicular transporter of glutamate, and vGAT, vesicular transporter of GABA in the two groups of rats. **(C)** Plots of glutamate and GABA intracellular levels assessed by Western blots of vGlut1 and vGAT, respectively. Note that both measures indicate an increase in glutamate levels [*t*_(1, 37)_ = 4.8; *p* < 0.0001] while GABA levels were reduced [*t*_(1, 21)_ = 2.4; *p* < 0.02] after MIUH, compared to sham rats. ^*^p < 0.05; ^***^p < 0.001.

To assess the balance between excitation and inhibition in the hind paw sensorimotor cortical area, we analyzed the contents of dialysates in extracellular glutamate and GABA obtained from P90 to P120 during *in vivo* microdialysis determined by gradient HPLC coupled to laser detection. The extracellular concentration of glutamate was greater in adult MIUH rats than in sham rats (*p* < 0.04); whereas, the extracellular concentration of GABA did not differ significantly between the two groups of rats (Figure 4A).

To complete microdialysis analysis, we performed semi quantitative Western blots in other adult animals, using specific antibodies against vesicular glutamate transporter (vGlut1) and vesicular GABA transporter (vGAT), reliable markers of excitatory and inhibitory transmission, respectively. The amount of vGlut1 increased of about 40% in MIUH rats relative to controls (*p* < 0.0001; Figures 4B,C). In contrast, the amount of vGAT decreased of about 15% (*p* < 0.02; Figures 4B,C). Thus, MIUH induces an increase in glutamate release in adult rats while GABAergic levels were reduced, suggestive of hyperexcitability in the hind paw area of the sensorimotor cortex.

## Discussion

This study is the first to show the early and enduring functional disorganization of neuromuscular interactions after MIUH in a rat model that recapitulates the diversity of the symptoms observed in children with encephalopathy of prematurity (6). MIUH in rats led to (1) reduced PAD at P4–P6, indicative of early hyperreflexia and spasticity, (2) an early reduction of the oligomeric (active) and monomeric (inactive) forms of KCC2, which regulates chloride homeostasis and cell excitability, (3) reduced PAD in adult rats, suggestive of enduring hyperreflexia and spasticity in adulthood, (4) changes in muscle contractile properties and phenotype in young-adults, and (5) cortical hyperexcitability in the adult sensorimotor cortex devoted to the hind limb representation.

### MIUH, hyperexcitability, and neuroinflammation

The presence of spasticity, hypertonicity, contractures or other related clinical signs is relatively common in children with CP (2) or in patients with stroke or spinal cord injury (SCI) (30–32) and has been reproduced in animal models of SCI (21–23). MIUH induced a decrease in PAD at P4–P6, a reliable correlate of early hyperreflexia and spasticity, as described in SCI rats (21–23). It is widely accepted that at least two mechanisms are responsible for such hyperreflexia after SCI: increased excitability in motoneurons and a reduction of inhibition, the so-called disinhibition, within the lumbar spinal network (21, 22). Disinhibition of the myotatic reflex seems to be caused by reduced expression of the active or oligomeric form of KCC2, which abnormally increases the intracellular concentration of chloride ions and reverses the effect of IPSPs from hyperpolarization to depolarization (21–23). In the present study, MIUH decreased the expression of inactive and active forms of KCC2, which induced early hyperreflexia and spasticity, like after SCI in neonate rats (21, 22). These results confirm and extend our previous study in which gastrocnemius histopathology indicated signs of spasticity (8). In addition, we also found a reduction of PAD in adult MIUH rats that suggests the persistence of increased stretch reflex, muscle hyperreflexia and likely spasticity in adulthood. It is worth noting that early and enduring increased stretch reflex and spasticity may be at the origin and persistence of the minor locomotor impairments found in adult MIUH (8).

It is well admitted that preterm infants and children with CP exhibit neuroinflammation processes from *in utero* to postnatal life (1, 5, 11, 17). MIUH induced early changes marked by the upregulation of several proteins related to inflammation and ischemic injury in placenta and the downregulation of mRNAs associated with axon and astrocyte growth in the fetal brains (7). As putative candidate, calpains, are intracellular proteases activated by calcium influx during glutamate-induced excitotoxicity and regulate cellular homeostasis, neuronal activity or apoptosis during development and in the mature CNS. Appropriate calpain activity is crucial for typical neurodevelopment, including learning and memory (33). Excessive calpain activity degrades proteins important for neural function (32), such as KCC2 in the hippocampus and layer IV of the cerebral cortex after MIUH at E18 (34) or in the spinal network after SCI (35). Indeed, recent studies have shown the deleterious contribution of excess calpain activity to cleave KCC2, which becomes inactive and thus contribute to the excitation/inhibition imbalance toward hyperexcitability (36) and to upregulate the persistent sodium current in motoneurons (37), thus leading to the development of hyperreflexia and spasticity, as shown after SCI (37, 38). Post-mortem cerebral samples from human preterm infants with WMI showed a loss of KCC2 expression (39).

Therefore, we suppose that MIUH-induced intrauterine inflammation (7) comes along with overactivity of calpains, which cleaves KCC2. Reduced expression of inactive (monomeric) and active (oligomeric) forms of KCC2 at P8 leads to hyperexcitability in the lumbar spinal cord, which likely contributes to postnatal hyperreflexia and spasticity, probably at the origin of the minor locomotion disorders observed later at P30 and P65 in MIUH rats (8).

### Enduring perturbations of neuromuscular interactions

To extend our results showing the early and enduring presence of hyperreflexia, spasticity and minor locomotor deficits, we investigated the contractile properties of the soleus muscle in young-adult MIUH rats to better understand the complex neuromuscular interplay after MIUH. At the muscle level, the most striking result was a drastic reduction in HRT in MIUH rats. Such a decrease has not been observed in plantarflexor muscles of children with spastic diplegia (40). The reduction of HRT suggests that following muscular contraction, the transport of calcium from the cytosol into the lumen of the sarcoplasmic reticulum by the sarco(endo)plasmic reticulum calcium ATPase (SERCA) is faster. This could be the result of a change in SERCA isoform expression toward fast isoforms (41). It could also be due to an increase in the activity of the SERCA pump. This latter hypothesis is sustained by the fact that an acute muscle ischemia results in an increase in the maximal SERCA activity (42). The modification of the HRT could also result from a proliferation of the sarcoplasmic reticulum. Indeed, a proliferation of the sarcoplasmic reticulum has been described in the soleus muscle of rats submitted to 2 and 4 weeks of hind limb unloading (43), as well as after denervation (44). Another parameter that influences the HRT is the muscle stiffness. Compliance accelerates relaxation in striated muscle by allowing myosin heads to move relative to binding sites on actin (45). However, this hypothesis is very unlikely since many papers have reported higher tissue stiffness in the triceps surae of patients with CP (46).

The other contractile kinetic parameters (TTP, P_20_/P_0_) were not modified. In adult rats, the P_20_/P_0_ ratio is near 80% for the soleus muscle, characteristic of a slow-type muscle. A value of 50% as observed herein shows that muscle maturation is still not achieved at P28. In addition, the persistence of neonatal MHC isoform suggests that MIUH delays the decline in expression of the neonatal isoform that occurs during typical development (47). A delay of maturation of the growth-associated shift of fiber phenotype toward slow type has also been observed in rats submitted to hypoactivity through hind limb unloading (48) or after denervation (47). These results emphasize the deleterious impact of abnormal motor commands in the maturation of muscle properties and in the expression of MHC isoforms (49). In the same way, HRT has been shown to decrease after deafferentation in rats, demonstrating that abnormal afferent inputs from muscle proprioceptors has in return an impact on muscle kinetic properties (27). Hyperreflexia and increased stretch reflex may occur as a compensatory process to abnormal somatosensory activity and muscle disuse (50). In fact, a reciprocal interplay exists between motoneuron firing, muscle properties/phenotype and proprioceptive feedback/reafference (27, 51) that contributes to the development and refinement of the sensorimotor circuitry.

**Figure 5:**
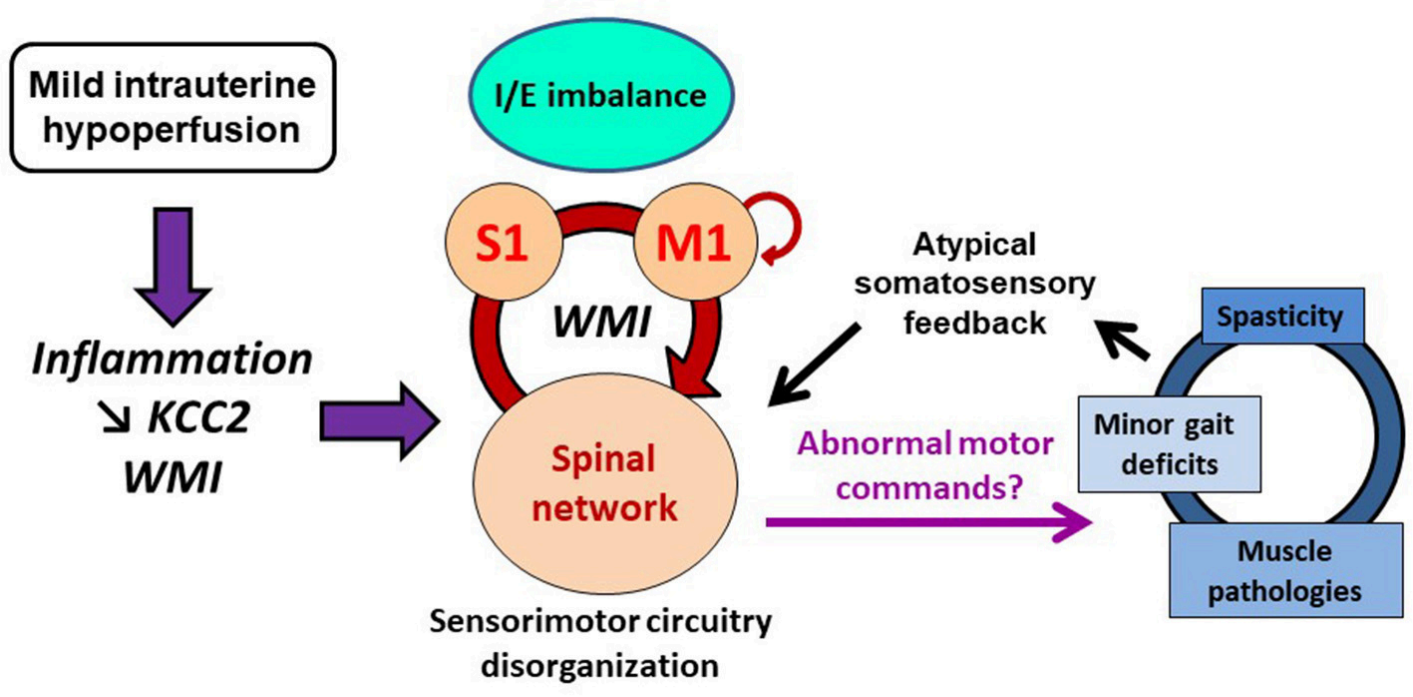
Schematic illustration of the possible pathophysiological cascade of mild intrauterine hypoperfusion (MIUH) on neuromuscular interactions and movements. First, MIUH induced early neuroinflammation, reduced KCC2 expression/function and white matter injury (WMI). These pathophysiological events led to early and long lasting neuroanatomical and functional disorganization of the sensorimotor circuitry, mainly involving spinal networks and the primary somatosensory (S1) cortex. Such a disorganization likely produced abnormal motor commands, resulting in muscle pathologies and overactivities, and locomotor impairments as well, which in turn provided atypical somatosensory feedback/reafference to the immature sensorimotor circuitry, maintaining or even aggravating its pathophysiological disorganization in a self-perpetuating cycle. I/E, inhibition/excitation; M1, primary motor cortex; S1, primary somatosensory cortex. Motor efferent copy to M1 is depicted by a small loop from/to M1.

In the hind limb area of adult sensorimotor cortex, MIUH induced an excitation/inhibition imbalance toward increased glutamate excitability while GABAergic inhibition was unchanged or slightly decreased in adult rats. Such a cortical imbalance may be the continuity and spread of the early disinhibition and hyperreflexia described at P8 in the spinal cord after MIUH. In fact, reduced KCC2 expression has been revealed in cortical layer IV at P8 and P28 in a comparable model of MIUH at E18 (52). This imbalance also corroborates our previous study in adult MIUH rats (9), in which we found a decrease in the inhibitory interneuron density in the primary somatosensory cortex and the degradation of both the neuronal properties and somatotopic maps, devoted to represent the hind limb skin surfaces. This degradation was mainly characterized by a reduction of the map size, enlarged and multiple receptive fields that encompassed several toes or pads simultaneously, leading to a somatotopic, and topographic disorganization (9). In addition, reduced KCC2 function/expression has also been evidenced to contribute to WMI (36), as previously observed in our MIUH model of EP (8–10). Since we found WMI below the somatosensory cortex, it is possible that early somatosensory inputs from the periphery were not spatiotemporally synchronized to induce appropriate spinal and cortical plasticity (6, 53). It is now widely accepted that WMI-induced changes in conduction velocity mediate abnormal transmission and integration of afferent and efferent information, leading to brain dysfunctions, as observed in several pathologies including autism and schizophrenia (54, 55). One can speculate that WMI below the somatosensory cortex may alter timing precision of somatosensory reafference arising from early spontaneous movements and locomotion to the immature sensorimotor circuitry, including the lumbar spinal cord, and sensorimotor cortex (Figure 5), associated with other subcortical structures (56). Because precision in timing of action potentials is fundamental in CNS plasticity (57, 58), early somatosensory inputs may have induced the postnatal and enduring disorganization within the sensorimotor circuitry after MIUH, described here and previously (6, 8–10, 59). Several studies have shown that abnormal processing and integration of afferent information in the somatosensory cortex is sufficient to drive disturbances in motor planning and execution (19, 60, 61).

Therefore, we speculate that early inflammation induced by MIUH at E17 led to reduced KCC2 expression and altered proliferation of oligodendrocyte precursors (1, 2, 3) that both contributed to postnatal WMI and lumbar hyperreflexia (Figure 5). Thus, postnatal WMI may induce sensorimotor circuitry disorganization, through inappropriate information synchrony and maladaptive plasticity (6, 53, 62, 63). In addition to lumbar hyperreflexia observed at P4–P6, such a disorganization of the sensorimotor circuitry from the spinal cord to the cortex may in turn produce abnormal motor commands, as suggested above in young-adult MIUH rats, which in turn may participate to induce and maintain altered muscle synergies, maturation and functional properties, as well as locomotor disturbances and musculoskeletal histopathology into a self-perpetuating cycle (Figure 5). In fact, we previously showed that postnatal movement restriction through transient hind limb immobilization during development induced musculoskeletal histopathology, locomotor disorders, and muscle overactivities including spasticity that interplayed into a self-perpetuating cycle, which likely contributed to maintain or even aggravate these disturbances (59). Thus, minor gait impairments related to MIUH likely produced atypical somatosensory feedback/reference to the immature sensorimotor circuitry that in turn participated to the functional disorganization of the sensorimotor circuitry in a second self-perpetuating cycle, while MIUH-related neuroinflammation contributed to the structural disorganization of the sensorimotor circuitry, including WMI (Figure 5).

### Functional implications

Early inflammation appears to be crucial in the pathogenic cascades and to contribute in the primary and secondary brain injuries, as well as in repair or recovery after insult events. Immunomodulatory interventions targeting inflammation seem beneficial in preclinical models and might have translational potentials (1, 5, 38). It appears crucial to develop new strategies to reinstate excitation/inhibition balance within the sensorimotor circuitry as early as possible after the insult and inflammation cascade inception. As a promising lead, erythropoietin (EPO) restores typical KCC2 expression in the hippocampus and brain following MIUH at E18 in rats (34, 36), and in motoneurons after neonatal stroke (64). In addition to neuroprotective properties (65), EPO modulates excess calpain activity via calpastatin (34, 36), reduces caspase activation (66), restores oligodendrogenesis, survival and process extension (67) after MIUH, and also improves motor recovery and neuronal regeneration after SCI (68). In addition, EPO is already used in clinical trials for many brain diseases including stroke, and in a clinical trial for extremely preterm infants (PENUT Trial).

Finally, our preclinical model based on MIUH contributes to elucidating the putative involvement of WMI on alterations in neural activity and plasticity, function, and refinement of the CNS. Further studies are required to enlighten the role WMI and altered neuromuscular interplay in the emergence of encephalopathy of prematurity and CP symptoms, particularly the development of NDDs such as ADHD and learning deficits, and to develop new strategies of prevention and rehabilitation.

## Author contributions

J-OC planned and contributed to all the experiments and wrote the manuscript. MD and NT-L performed *in vivo* microdialysis. FrC performed western blotting in the sensorimotor cortex. LB, PC, FloC, CB, and SL performed western blotting of KCC2. VM, BB, and M-HC assessed the contractile properties. JP, FloC, and J-OC performed early and adult PAD. YO, MT, and J-OC assessed intrauterine blood flowmetry. J-OC, M-HC, and FloC performed all statistics. J-OC, MD, CB, SL, BB, MT, M-HC, and FloC contributed to write, edit and revise the manuscript.

### Conflict of interest statement

The authors declare that the research was conducted in the absence of any commercial or financial relationships that could be construed as a potential conflict of interest.
