# Supplementary material for: Mild Intrauterine Hypoperfusion Leads to Lumbar and Cortical Hyperexcitability, Spasticity, and Muscle Dysfunctions in Rats: Implications for Prematurity
Source: Front Neurol. 2018 Jun 15;9:423. doi: 10.3389/fneur.2018.00423 (PMC6020763; doi:10.3389/fneur.2018.00423)
Supplement: Supplementary file 1 [file Data_Sheet_1.DOCX]

**Supporting Information**

**Title: Mild intrauterine hypoperfusion leads to lumbar and cortical hyperexcitability, spasticity and muscle dysfunctions in rats: implications for prematurity**

Jacques-Olivier Coq^ab*^, Maxime Delcour^b†^, Yuko Ogawa^c^, Julie Peyronnet^a^, Francis Castets^d^, Nathalie Turle-Lorenzo^e^, Valérie Montel^f^, Laurence Bodineau^g^, Phillipe Cardot^g^, Cécile Brocard^a^, Sylvie Liabeuf^a^, Bruno Bastide^f^, Marie-Hélène Canu^f^, Masahiro Tsuji^c^, Florence Cayetanot^a,g^

**^a^** Institut de Neurosciences de la Timone, UMR 7289, CNRS, Aix Marseille Université, 13385 Marseille, France

^b^ Neurosciences Intégratives et Adaptatives, UMR 7260, CNRS, Aix Marseille Université, 13331 Marseille, France

^c^ Department of Regenerative Medicine and Tissue Engineering, National Cerebral and Cardiovascular Center, Suita, Osaka 565-8565, Japan

**^d^** Institut de Biologie du Développement de Marseille, UMR 7288, CNRS, Aix-Marseille Université, 13288 Marseille, France

**^e^** FR 3512 Fédération 3C, Aix Marseille Université – CNRS, 13331 Marseille, France

**^f^** Université de Lille, EA 7369 « Activité Physique, Muscle et Santé » - URePSSS - Unité de Recherche Pluridisciplinaire Sport Santé Société, 59000 Lille, France

^g^ Sorbonne Université, Inserm, UMR_S1158 Neurophysiologie Respiratoire Expérimentale et Clinique, 75005 Paris, France

1. **Supplementary Methods**
2. **Supplementary Figure 1**
3. **Supplementary Figure 2**
4. **Supplementary Methods**

**Excitatory and inhibitory neurotransmission**

***In vivo microdialysis****.* Carnegie Medecin microdialysis probes (CMA/11, Phymep, France) were implanted within the hindpaw representation of the right S1-M1 area. Each rat was dialysated two hours without collecting, in order to reach a basal neurotransmitter level after the introduction of the probe. Dialysis probe was perfused at 2 µL.min^-1^ using a microinjection pump (CMA/100, Phymep), with an artificial CSF containing (in mM): NaCl = 147; KCl = 2.7; CaCl2 = 1.2; MgCl2 = 0.85 (CMA, Phymep). Dialysates were collected every 5 min (10 µL by sample) on a Univentor refrigerate microfraction collector (Phymep) during 30 min and stocked at –80 °C before analysis.

The extracellular concentrations of glutamate (GLU) and GABA in the dialysates were determined by gradient High Performance Liquid Chromatography (Dionex, France) coupled with laser detection (Picometrics, France). Pre-column derivation consisted in a NDA (2,3Naphthalenedicaboxaldehyde) derivation. Thirty min before analysis, a solution F freshly synthesised (20 µL) containing 1.6 mL of Solution E (in 100 mL of distilled water) = Sodium tetraborate + Boric Acid; 400 µL of Solution D (in 10 mL of distilled water) = 28 mg Potassium Cyanide and 333 µL of Solution C (in 10 mL of distilled water and acetonitrile in the same proportion) = 9.2 mg of NDA was added to each sample (10 µL). During these 30 min, the samples were maintained at room temperature, in the dark. Following these procedures, 8 µL of the 30 µL derived sample were injected into a C18 column (300 µm x 150 mm, i.d.; particle size 3 µm; Dionex) eluted with buffer 1 which contained citrate sodium 0.05 adjusted to pH 3.5 and buffer 2 containing 100% Acetonitrile. The concentration gradient was established for the best separation of GLU and GABA. The retention time was 13.5 min and 19.5 min for GLU and GABA, respectively. The column was perfused at 0.7 mL.min^-1^ and maintained at 25°C. Detection was performed by a laser (LIF, Picometrics) and the signal was integrated using a Dionex Integration Pack.

***Western blotting and quantification.*** Western blot quantification was performed on autoradiography with Image J software (Abramoff et al., 2004). Integrative intensities minus background were plotted for each sample after normalization to 1 for the highest value for one western blot.

Mouse monoclonal anti-tubulin (E7) antibody, from the Developmental Studies Hybridoma Bank (Iowa city, IA, USA), was used at 0.2 µg.mL^-1^. Mouse monoclonal anti-vGLUT1 antibody (clone N28/9, NeuroMab, UC Davis, CA) was used at 1 µg.mL^-1^. Rabbit polyclonal anti-VGAT antibody (#AB5062P Millipore, Temecula, CA) was used at 1µg.mL^-1^.

**Muscle contractile properties and wester blotting**

Muscle contractile phenotype was determined through analysis of MHC composition. Myofibrillar proteins were extracted from muscle powder in a buffer containing 20 mM Tris, pH 7.4, 4 mM EGTA, 10 mM EDTA containing anti-proteases (Complete EDTA-free, Roche Diagnostic) as presviously described (Toursel et al., 2000), washed and resuspended in 500 μl of milliQ water. Their concentration was determined. The proteins were then precipitated for 2 h with acetone, followed by centrifugation for 1 h at 13,000 rpm. The pellet was dissolved in Laemmli solution for SDS–PAGE. Protein estimation of supernatant was done using Bradford

assay (Bio-Rad). The MHC composition was determined by SDS-PAGE on a 7.5% separating polyacrylamide gel. Electrophoresis was run for 22 h at 12 °C (180 V constant , 13 mA per gel). Then the gels were sensitized with glutaraldehyde and silver stained. GS-800 Imaging densitometer and QuantityOne Software (Bio-Rad, Hercules, CA) were used to determine the relative proportions of the different MHC isoforms in each muscle. Slow (MHC I) and fast (MHC IIa, IId/x, and IIb) isoforms were identified according to their migration and previous report of the laboratory. The neonatal MHC isoform was identified using immunoblot analysis with a monoclonal antibody.

After separation, proteins were transferred to 0.45 μm nitrocellulose membranes (Hybond, GE Healthcare) using transfer buffer (20 mM Tris base; 150 mM glycine; 20 % methanol), added with 0.025 % SDS for the transfer of high-molecular weight proteins. Proteins load and quality of transfer were verified by Ponceau staining. The blots were then washed in TBST (15 mMTris/HCl, pH 7.6; 140 mMNaCl; 0.05 % Tween-20) and blocked in 5 % non-fat dry milk in TBST. Membranes were then blotted with primary antibody against neonatal MHC ( ) overnight at room temperature (RT) at 4 °C. Membranes were washed 3×10 min in TBST, and then incubated with HRPlabelled secondary antibodies in blocking solution for 2 h at RT followed by 5×10 min washes in TBST. Chemiluminescence detection was carried out using ECL Clarity (Biorad), and images capture were done with ChemiDoc MP (Biorad). All the images were analyzed using the Image Lab 4.0.1 software (Biorad)

1. **Supplementary Figure 1**

Total protein level after gel loading and transfer to nitrocellulose membrane. The total protein amount was detected by Stain-Free technology after transfer. Stain-Free gels (Bio-Rad) contain a trihalocompound within the gel matrix that produces a fluorescent product when covalently crosslinked to protein tryptophan residues. To activate the stain-free gel, the gel was exposed to UV light for 1 minute using Chemidoc XRS (Biorad) imaging system equipped with a UV illumination source. Immediately after the transfer was completed, protein bands have been detected using the same imaging system.

**A** Sham MIUH

**
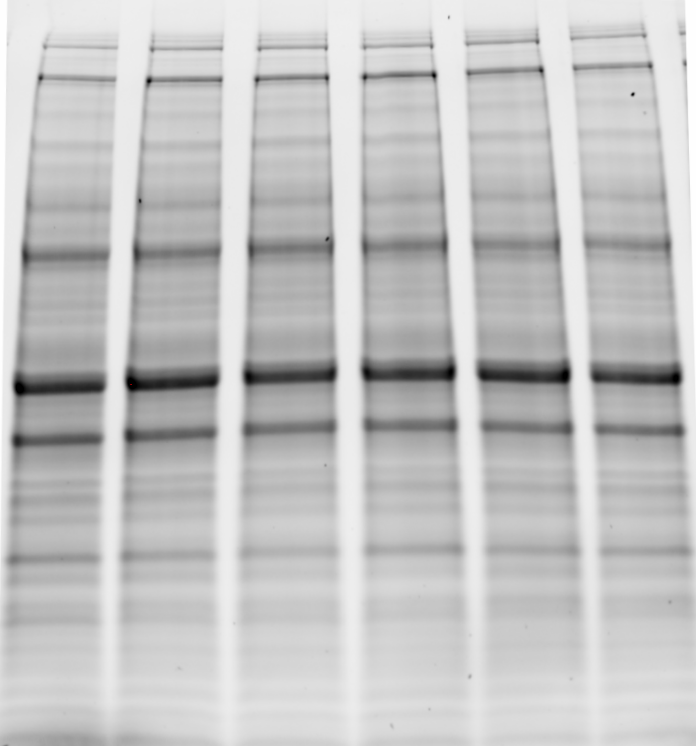
**

1. **Supplementary Figure 2**

Original pictures used to construct the figure 4B and C. **A.** Ponceau red staining of nitrocellulose membrane used for vGlut1 and tubulin revelation. **B.** Original scan of the film obtained with vGlut1 antibodies. **C.** Original scan of the film obtained with tubulin antibodies. **D.** Ponceau red staining of nitrocellulose membrane used for vGAT revelation. **E.** Original scan of the film obtained with VGAT antibody.

**A** **D**


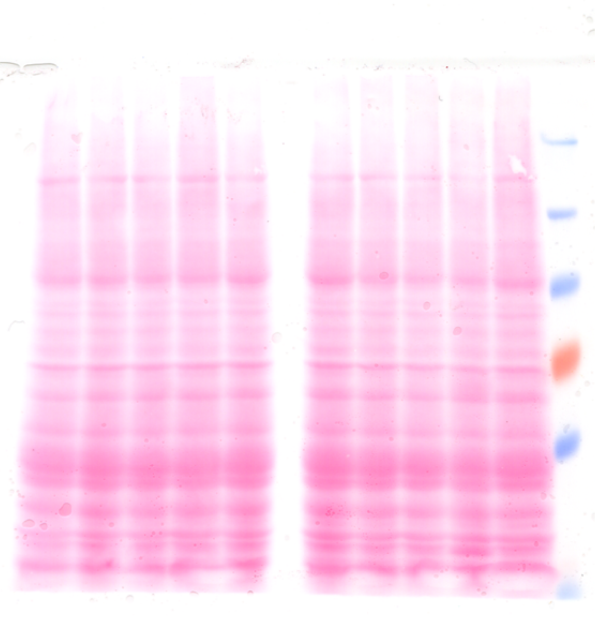
Sham MIUH Sham MIUH

**
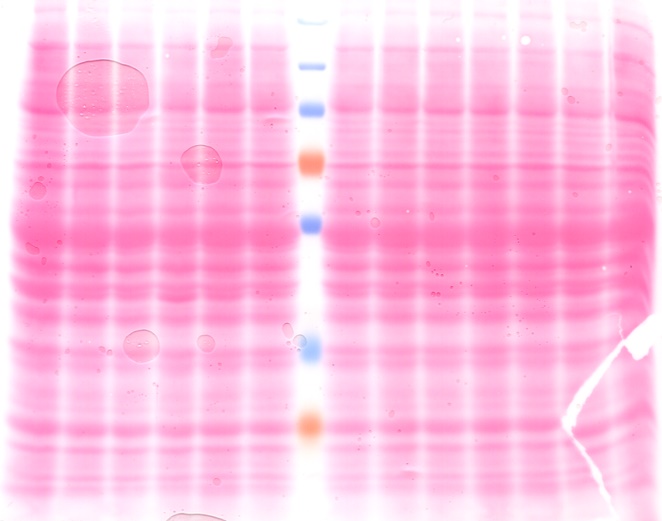
**

**B**


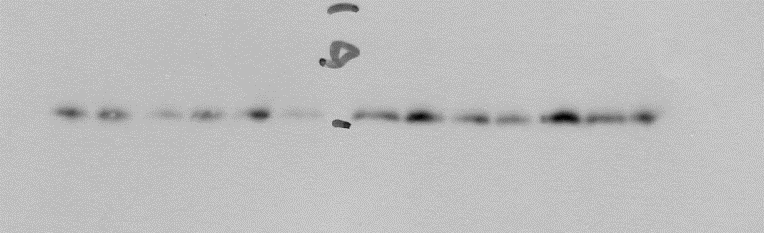


**C**


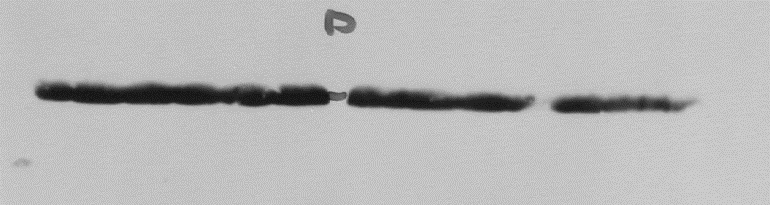


**E**


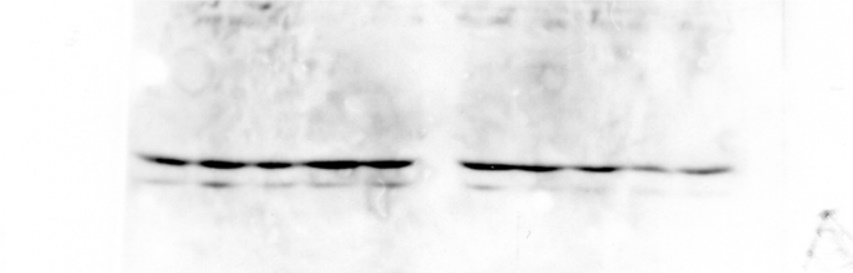


**References**

Abramoff MD, Magalhães PJ, Ram SJ (2004) Image processing with ImageJ. Biophotonics Int 11:36–42.

Toursel T, Bastide B, Stevens L, Rieger F, Mounier Y (2000) Alterations in contractile properties and expression of myofibrillar proteins in wobbler mouse muscles. Exp Neurol 162:311–320.
